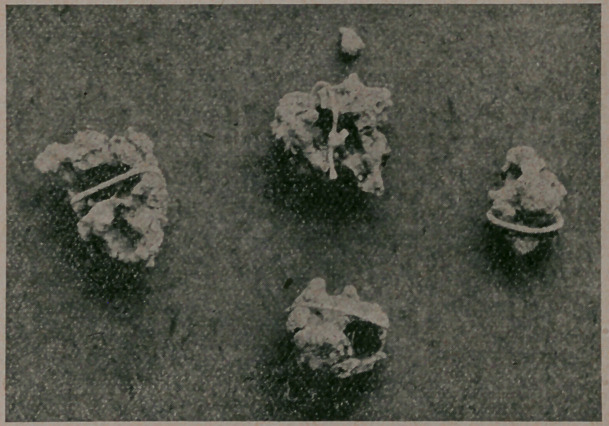# Unilateral Salpingo Oophorectomy and Appendicectomy, with Removal of Four Calculi from the Transverse Meso-colon

**Published:** 1898-06

**Authors:** B. F. Kingsley

**Affiliations:** San Antonio, Texas


For the Texas Medical Journal.

### Unilateral Salpingo Oophorectomy and Appendicectomy, with Removal of Four Calculi from the Transverse Meso-colon.

BY B. F. KINGSLEY, M. D., SAN ANTONIO, TEXAS.

[Read at the Houston Meeting of the Texas State Medical Association, April 28, 1898.]

Mrs. K., German, age 33, multipara. First seen by me November 6th, 1896. She had had a normal confinement two months previously, followed in about ten days with pain in lower abdomen, especially in the right side, for which she was compelled to remain in bed most of the time. She had frequent paroxysms of sharp pain extending into the right scapular region and shoulder, while there was marked tenderness on pressure in the region of the gall bladder, as well as over the appendix and ovary. In order to get any ease she had to lie in bed with her right leg flexed on the abdomen, any change from this position causing excessive pain. Digital examination of the womb revealed great tenderness in the right broad ligament, with marked tension and immobility of the tissues and womb on the right side and a temperature of 100° F. An operation was advised and done on November 12th. The right tube and ovary were considerably enlarged and inflamed, the broad ligament thickened with adhesions everywhere. These were released, the tube and ovary removed, the appendix found behind the cœcum and very adherent. It was dissected out and found to contain no mesentary of usual length, but greatly enlarged and inflamed. It was removed and the peritoneum closed over the stump. In exploring the upper abdomen for an explanation for the scapular pains, thinking I might perhaps find some gall stones, to my surprise I found some hard and sharp nodules, which were turned out, and proved to be four calculi imbedded in the transverse meso-colon. They were dissected out and wounds closed with fine silk sutures, each wound sponged dry, and the abdomen closed. Her recovery was rapid, and she left the hospital on the sixteenth day, and has been perfectly well since. Nursing was suspended for a few days until all danger from peritonitis was over, when it was resumed, the breast being relieved in the meantime by the pump.

The case is interesting chiefly from two standpoints. First, the etiology which is involved in uncertainty whether the inflammation originated in the appendix or tube and ovary, there being little doubt about its infectious character; and second, peculiar location and formation of the calculi, the only instance of the kind I have seen in many abdominal operations.

San Antonio, Texas, April 18th, 1898.

## Figures and Tables

**Figure f1:**